# Divergence, demography and gene loss along the human lineage

**DOI:** 10.1098/rstb.2010.0004

**Published:** 2010-08-27

**Authors:** Hie Lim Kim, Takeshi Igawa, Ayaka Kawashima, Yoko Satta, Naoyuki Takahata

**Affiliations:** 1 Hayama Center for Advanced Studies, Hayama, Kanagawa 240-0193, Japan; 2 Department of Evolutionary Studies of Biosystems, Hayama, Kanagawa 240-0193, Japan; 3 The Graduate University for Advanced Studies (Sokendai), Hayama, Kanagawa 240-0193, Japan

**Keywords:** primates, modern humans, ancestral polymorphism, pseudogenes

## Abstract

Genomic DNA sequences are an irreplaceable source for reconstructing the vanished past of living organisms. Based on updated sequence data, this paper summarizes our studies on species divergence time, ancient population size and functional loss of genes in the primate lineage leading to modern humans (*Homo sapiens sapiens*). The inter- and intraspecific comparisons of DNA sequences suggest that the human lineage experienced a rather severe bottleneck in the Middle Pleistocene, throughout which period the subdivided African population played a predominant role in shaping the genetic architecture of modern humans. Also, published and newly identified human-specific pseudogenes (HSPs) are enumerated in order to infer their significance for human evolution. Of the 121 candidate genes obtained, authentic HSPs turn out to comprise only 25 olfactory receptor genes, four T cell receptor genes and nine other genes. The fixation of HSPs has been too rare over the past 6–7 Myr to account for species differences between humans and chimpanzees.

## 1. Introduction

The last two decades have witnessed explosive advances in molecular evolutionary studies that are based on a large amount of DNA sequence information. Darwin's dream of reconstructing the tree of life has come true and much light has been thrown on the origin of man and its history (Darwin 1859, 1871).

Using all the available DNA sequences as of 2009, we review our genetics studies on primates with special reference to the origin and demographic history of modern humans (*Homo sapiens sapiens*). Section 2 addresses the species divergence time and ancient population size of six primate species. Two main conclusions are drawn regarding the rather ancient divergences of major primate taxa and rather large ancestral population sizes. Section 3 is concerned with the origin of modern humans. To distinguish between two alternative hypotheses for their origin, we re-examine DNA polymorphism data on 37 loci in the three major ethnic groups. At individual loci, we determine the most ancient type of genes in a sample, the time to the most recent common ancestor (TMRCA), and the place or group in which the most ancient type of genes occurs most frequently (PMRCA).

Section 4 enumerates human-specific pseudogenes (HSPs), in order to understand their role in human evolution and relationships to palaeo-environments. Unexpectedly, authentic HSPs are more limited than presently claimed, thereby bringing into question the functional loss of genes as a major driving force in human evolution. Finally, a short perspective is given on human evolutionary genetics.

## 2. Primate divergence and demography

Except for the extreme conditions that may be found with endangered species, any bisexual diploid species is almost always genetically polymorphic. The larger the effective population size (*N*_e_), the more ancient the origin of the polymorphism. DNA sequences at a locus chosen from a population are necessarily derived from the most recent common ancestor (MRCA), in the absence of recombination. Owing to randomness in the reproduction process, the time (**τ**) at which a randomly selected pair of alleles at a locus can be traced back to the MRCA is a random variable. Under selective neutrality (Kimura 1968), the probability distribution of **τ** is exponential with the average value of 2*N*_e_ generations (Kingman 1982). If this species splits into two populations, both must initially inherit more or less the same set of polymorphisms that were present in the ancestral species. As time elapses, the descendant populations gradually differentiate from each other and evolve into new reproductively isolated species. In *t* years or *t*/*g* generations (with a generation time of *g* years) after the populations split from each other, the extent of the inherited ancestral polymorphism at a given locus decreases and, eventually, only one ancestral gene lineage remains in each descendant species. Of course, this does not necessarily mean that these descendant species are genetically monomorphic, since new mutations continuously accumulate and cause differentiation from the ancestral gene lineage.

For orthologous gene pairs at different loci sampled from two extant species with a divergence time *t*, we can observe a set of the number (*k*) of nucleotide differences per site that have accumulated at each locus since the MRCA **τ*g* + *t* years ago. The value of *k* differs from locus to locus and is governed by the probability laws for the coalescence time **τ** and the stochastic nature of accumulating nucleotide substitutions in a gene lineage. For a given set of DNA sequence data for a pair of species, we have developed a maximum-likelihood (ML) method to infer *t* and ancestral *N*_e_ (Takahata *et al*. 1995). In this method, *t* and *N*_e_ are scaled by the nucleotide substitution rate (**μ**) per year per site such that *y* = 2*t*μ** stands for the net nucleotide difference between the two extant species and *x* = 4*N*_e_*g*μ** stands for the nucleotide diversity in the ancestral species.

Since the ML method was originally based on several simplified assumptions, Yang (1997) extended to the case where the rate of nucleotide substitutions may differ among loci. Yang (2002) and Rannala & Yang (2003) further developed the Markov chain Monte Carlo (MCMC) method for the more general case where more than two extant species are included in a sample and the number of DNA sequences may differ among loci. While the current MCMC method cannot be applied to synonymous sites, it permits us to use other types of DNA sequence data at multiple loci from multiple extant species simultaneously.

The MCMC method was previously applied to 53 intergenic sequence data from four primate species (Chen & Li 2001). The method yielded smaller estimates of *N*_e_ (Rannala & Yang 2003) than the ML for synonymous sites (Takahata & Satta 1997; Takahata 2001). The small MCMC estimates may be attributable to the nature of the data because the ML method also gave rather small estimates of ancestral *N*_e_ for the same data (Satta *et al*. 2004). Nonetheless, it is instructive to note the strong dependence of MCMC estimates on the prior distribution. The posterior mean tends to be confined to local areas near a given prior mean if the prior standard deviation (s.d.) is assumed to be small. In the opposite case of a large prior s.d., the posterior mean tends to differ greatly from the prior mean, whereas the posterior s.d. becomes correspondingly large. We tested if the previous result in Rannala & Yang (2003) is robust to the prior distribution. Our tentative conclusion for the MCMC method is that we must assume that the prior s.d. is no smaller than the prior mean.

With this in mind, we used both the ML and MCMC methods to re-examine autosomal DNA sequence data available for six primate species (electronic supplementary material, figure S1). The data include 53 intergenic sequences (Chen & Li 2001) together with additional orthologous sequences from Old and New World monkeys, 17 intron sequences (O'hUigin *et al*. 2002) and 13 intergenic sequences newly retrieved from databases (electronic supplementary material, table S1). In total, we used 83 loci and to our knowledge, this is the largest dataset to be analysed with the inclusion of six primate species.

There are two exceptionally large datasets—the 58 Mb BAC end sequences (BES) for humans and chimpanzees (Fujiyama *et al*. 2002) and 18 Mb shotgun sequences for humans, chimpanzees, gorillas and macaques (Patterson *et al*. 2006). We exclude these datasets from the present analysis, mainly because they were thoroughly examined in Satta *et al*. (2004) and Burgess & Yang (2008), respectively, and because the number of primate species studied was four at most.

The ML or MCMC method yielded estimates of *y* (or *y*/2) and *x* for six primate species (table 1). It is clear that the ML estimates are more similar to the MCMC estimates for the second set of broader priors than the first set. To convert *y* and *x* to *t* and *N*_e_, we must know the nuisance parameters **μ** and *g*. It has long been argued that the nucleotide substitution rate has slowed down in hominoids and that the generation time as a life-history trait has been greatly lengthened in human evolution (Bogin 2009). If we assume that the human and the orangutan diverged 14 Myr ago, **μ** becomes a little smaller than 10^−9^. With this estimate, both methods roughly estimate the 30 Myr separation time between hominoids and Old World monkeys and the 45 Myr separation time between hominoids and New World monkeys (cf. Kumar & Hedges 1998; Takahata 2001). However, for the human and chimpanzee divergence to be at least 6 Myr ago, **μ** in this hominoid dataset must be as small as 0.7 × 10^−9^, which is consistent with the rate slow-down hypothesis.

**Table 1.** The ML and MCMC estimates (%) of *y*/2 = *t*μ** and *x* = 4*N*_e_*g*μ** based on 83 loci sampled from humans (H), chimpanzees (C), gorillas (G), orangutans (O), Old World monkeys (M) and New World monkeys (N). In the MCMC estimates, all species specified by subscripts are used, whereas in the ML estimates, H and the most distantly related species specified by the subscripts are used. See electronic supplementary material, table S1 and figure S1 for detail.

		MCMC1^a^		MCMC2^a^	
	ML	prior-1	posterior-1	prior-2	posterior-2
*x*_HC_	0.35	0.10 ± 0.10	0.27 ± 0.11	1.00 ± 1.00	0.43 ± 0.19
*x*_HCG_	0.39	0.10 ± 0.10	0.38 ± 0.06	1.00 ± 1.00	0.39 ± 0.06
*x*_HCGO_	0.52	0.10 ± 0.10	0.24 ± 0.12	1.00 ± 1.00	0.36 ± 0.10
*x*_HCGOM_	1.03	0.10 ± 0.10	0.55 ± 0.12	1.00 ± 1.00	0.74 ± 0.16
*x*_HCGOMN_	2.73	0.10 ± 0.10	1.54 ± 0.24	1.00 ± 1.00	2.39 ± 0.40
*y*/2_HC_	0.41	0.50 ± 0.11	0.45 ± 0.03	0.50 ± 0.11	0.42 ± 0.04
*y*/2_HCG_	0.53	0.66 ± 0.15	0.55 ± 0.03	0.66 ± 0.15	0.55 ± 0.03
*y*/2_HCGO_	1.23	1.40 ± 0.37	1.40 ± 0.06	1.40 ± 0.37	1.35 ± 0.05
*y*/2_HCGOM_	2.42	3.00 ± 0.60	2.65 ± 0.08	3.00 ± 0.60	2.57 ± 0.09
*y*/2_HCGOMN_	4.00	5.00 ± 0.80	4.59 ± 0.15	5.00 ± 0.80	4.35 ± 0.16

^a^Two sets of prior mean and standard errors are examined.

On the other hand, the estimate of *x* for the hominoid ancestor is about 0.4 per cent, which is five times larger than the extent of the DNA polymorphism in the extant human population (e.g. Li & Sadler 1991; Yu *et al*. 2002; Nachman *et al*. 2004; Zhao *et al*. 2006). In addition, the generation time in the hominoid ancestor is likely to have been 10 years, suggesting that *N*_e_ = 10^5^ rather than 10^4^, as in the extant human population (Takahata 1993; Takahata *et al*. 1995).

We are concerned about the possibility that our large estimates of *x* in the case of large *y* values (table 1) may result from computational problems. By computer simulation with 100 loci, we found that both ML and MCMC methods can recover the assumed values reasonably well even in the case where *x* is as small as 0.04 per cent and *y* is as large as 20 per cent. Thus, the large *N*_e_ in the early primate ancestor does not appear to be a computational artefact.

## 3. Modern human demography

After splitting from the chimpanzee lineage 6–7 Myr ago, the human lineage has undergone significant changes in morphology, physiology and behaviour (Leakey 1994). Before the emergence of the genus *Homo*, a number of hominid speciation events occurred in Africa in the Pliocene. Something unusual took place about 2 Myr ago, around which time *Homo erectus* migrated from Africa to Southeast Asia. The second *Out-of-Africa* event took place much later, involving modern humans that had spread over the world by 20 000 years ago. The origin of modern humans has long been debated, particularly with respect to the possibility of interbreeding between the expanding modern humans and the original inhabitants (Cann *et al*. 1987; Takahata 1993; Wolpoff *et al*. 2000; Takahata *et al*. 2001; Klein & Takahata 2002; Satta & Takahata 2002; Templeton 2002).

In our dataset, the present human population is subdivided into three major groups, consisting of Africans (Af), Europeans (Eu) and Asians (As). The Hispanic population sample, genotyped in the National Institute of Environmental Health Sciences (NIEHS), is treated separately, although it can be regarded as an admixture group between Europeans and descendants of Asians (Amerinds). The pattern and extent of DNA polymorphisms differ from one group to another for historical reasons.

Previously, Takahata *et al*. (2001) examined 10 X-chromosomal loci, five autosomal loci, mitochondrial DNA (mtDNA) and one Y-chromosomal locus (YAP). The TMRCA ranges from about 0.2 Myr for haploid mtDNA and YAP to 1.6 Myr for both X-chromosomal and autosomal loci, whereas the PMRCA is mostly assigned to Africans. Incidentally, TMRCA or the time scale of DNA polymorphism in living human populations encompasses that of the entire history of the genus *Homo*. DNA polymorphism thus reflects the demographic history of *Homo*. In particular, PMRCA contains information about relative population sizes or different population structures for the three major groups and the lengths of their histories. If one group has dominated in these respects, it is likely that the PMRCAs for individual loci are unevenly distributed among the groups. However, the sample size or the length of DNA sequences was not sufficiently large at some loci. Subsequently, more DNA polymorphism data have been accumulated, yielding more reliable estimates.

Here, based on the maximum-parsimony method for estimating the MRCA sequence in a human sample with one chimpanzee orthologue, we re-examine the TMRCAs and the PMRCAs at 37 loci with each having a worldwide sample of greater than or equal to 60 chromosomes (table 2). Of these loci, 18 are previously reported and the remaining 19 come from randomly retrieved NIEHS genotype data from which haploid sequences are inferred. The estimated TMRCAs for autosomal and X-linked loci range from 0.3 Myr at PLCG1 to 3.1 Myr at APOE. The average TMRCA at the 31 autosomal loci alone becomes 1.24 Myr, if humans and chimpanzees diverged 6 Myr ago. The extant polymorphisms at most loci in the human population were thus generated in the Pleistocene period. Some exceptions are EDN, CMAH, ASAH1, CD209, APOE and RRM2P4 loci, at which the TMRCA is greater than 2 Myr. Since there are no such loci among the 19 loci derived from NIEHS single nucleotide polymorphism data, there might be some bias towards reporting highly polymorphic loci in the literature. In any event, such a high proportion of six out of 31 autosomal loci (19%) with a TMRCA greater than 2 Myr may indicate a significant demographic change in the human population during the Pleistocene.

**Table 2.** TMRCA and PMRCA at 37 genomic regions. The results of the first 10 loci are taken from Takahata *et al*. (2001) and Satta & Takahata (2004) and those of the next eight loci are taken from Hayakawa *et al*. (2006), Zhao *et al*. (2006), Kim & Satta (2008), Patin *et al*. (2006), Barreiro *et al*. (2005), Fullerton *et al*. (2000), Cox *et al*. (2008) and Yu *et al*. (2002).

regions	chromosome	length (bp)	sample size	TMRCA (Myr)^a^	PMRCA^b^
HFE	6	11 214	60	1.08	Af
HBB	11	2998	264	1.63	Af
ECP	14	1203	108	0.51	Af
EDN	14	1214	134	3.03	Af
MC1R	16	954	242	0.85	Af
HBA	16	350	276	1.43	Af
ZFX	X	1215	335	1.21	Af
Xq13.3	X	10 163	69	0.67	Af
MAOA	X	4260	146	1.43	Af
mtDNA	mt	610	189	0.20	Af
CMAH	6	7302	132	2.90	Eu
6p22	6	12 113	122	0.60	Af
ASAH1	8	4358	60	2.40	Af
NAT1	8	2605	160	2.01	As
CD209	19	5500	254	2.80	Af
APOE	19	5491	192	3.11	Af
RRM2P4	X	5667	253	2.33	Af
DACH2	X	10 346	62	1.20	Af
ENO1	1	6165	174	0.33	Af
MAD2L2	1	5018	172	0.59	Af
ODC1	2	8003	174	1.00	Af
ATOX1	5	7546	168	0.08	Af
MAPK9	5	6780	176	0.70	Af
RAD1	5	7684	174	1.19	Af
SEPP1	5	10 108	174	0.43	Af
VNN3	6	7684	156	0.93	Af
MSH5	6	4745	148	0.48	Af
PEO1	10	8598	172	0.59	Eu
PRDX3	10	11 316	140	0.78	Af
CSK	15	8586	166	0.69	Hs
DUT	15	11 453	164	1.00	Af
TGFB1I1	16	6719	164	0.67	Af
EPX	17	7549	166	0.47	Af
PLCG1	20	11 039	170	0.32	Af
SPO11	20	11 724	150	1.01	Af
GABPA	21	5851	172	1.09	Af
TBX1	22	4488	178	0.77	Af

^a^Estimates are either taken from the original papers or made based on the assumption of the 6 Myr divergence time between humans and chimpanzees.

^b^‘Af’, ‘Eu’, ‘Hs’, and ‘As’ stand for Africans, Europeans, Hispanics, and Asians, respectively. For instance, ‘Af’ indicates that the ancestral haplotype is most frequent in Africans.

In fact, since the average TMRCA is roughly equal to 4*N*_e_*g* years under neutrality, *N*_e_ becomes 1.55 × 10^4^ from the observed average TMRCA = 1.24 Myr and *g* = 20. There are also other statistics for estimating *N*_e_ from polymorphism data. One is the number (*s*) of segregating sites per site (Watterson 1975). With the average *s* value being 0.11 per cent in our sample, we can estimate *N*_e_ as 1.40 × 10^4^ from Watterson's formula and the assumption of **μ** = 10^−9^ per site per year. Thus, *N*_e_ becomes about 1.5 × 10^4^ in both estimates. If **μ** is as small as 0.7 × 10^−9^ as mentioned earlier, the *N*_e_ values become correspondingly large. These estimates of *N*_e_ are at least 1.5 times greater than the previous estimate of 10^4^ (Takahata 1993) but smaller than 10^5^ for the common ancestral population of humans and chimpanzees as mentioned in §2.

**Figure 1.**
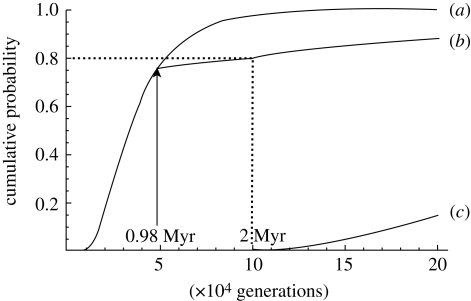
The probability of TMRCA smaller than *t* for a sample of 60 DNA sequences at a locus; see eqn (7) in Takahata & Nei (1985) and note that the exponent in eqn (7b) should contain a minus sign. The curves (*a*), (*b*) and (*c*) represent the case of *N*_e_ = 10^4^ throughout, the case of *N*_e_ = 10^4^ for *t* < *t*_b_ and 10^5^ for *t* ≥ *t*_b_ where *t*_b_ = 0.98 Myr, and the case of *N*_e_ = 10^5^ throughout, respectively. The generation time is assumed to be 20 years.

One of us suggested a one-order reduction in population size during the Pleistocene or a Pleistocene bottleneck in human evolution (Takahata 1993). Actually, under a demographic model of a constant *N*_e_ = 10^4^, the probability that 60 genes sampled for a locus coalesce to the MRCA within the past 2 Myr or 10^5^ generations is as high as 0.98 (Takahata & Nei 1985). On the other hand, if *N*_e_ = 10^5^, the same probability becomes as small as 0.004. For simplicity, we assume a sudden Pleistocene bottleneck model with *N*_e_ = 10^5^ before *t*_b_ years and *N*_e_ = 10^4^ after *t*_b_ years. We then determine the most likely value of *t*_b_, for TMRCA greater than 2 Myr to occur among 19 per cent of the loci. The *t*_b_ value thus estimated is 0.98 Myr (figure 1) and suggests that the bottleneck occurred during the Middle Pleistocene. The subsequent population expansion in the Upper Pleistocene and Holocene is too recent to alter the conclusion in any significant way.

The PMRCA analysis indicates that, in 33 of the 37 cases (89%), Africans possess the most ancient type of genes, whereas non-Africans generally possess derived types of genes. Africans have thus maintained about eight times more distinct gene lineages than non-Africans. This PMRCA or lineage asymmetry may be attributed to an extremely large effective size or a more subdivided population structure of Africans relative to non-Africans. However, since it is unrealistic to assume that the effective size of the entire non-Africans was as small as 10^3^, the African subdivision hypothesis is more likely. In this scenario, a necessary condition is the existence of some African subpopulations that have not directly exchanged migrants with non-Africans (Satta & Takahata 2002, 2004) and that could retain ancestral types of genes. It appears that no comparable subpopulation structure has existed in Eurasia, even though *H. erectus* occupied the area and inherited correspondingly ancient types of genes.

Modern human descendants migrating out of Africa might have encountered and interbred with former *H. erectus* inhabitants. Our PMRCA analysis suggests that genes that were maintained in Africa and that spread over Eurasia have by and large swamped those genes that were inherited by descendants of *H. erectus*. There is little or no strong genetic signal for multi-regional origins of modern humans (Wolpoff *et al*. 2000).

## 4. Functional loss of genes

Olson (1999) argued that functional loss of genes can frequently occur by means of numerous molecular causes and proposed the *less-is-more* hypothesis. The hypothesis is based on the observation that a large fraction of genetic functions of a genome are dispensable and on the speculation that selection may permit emergence of a less complete genome. Likewise, one of us (Takahata 1999) emphasized that dispensability of genes should be taken as evidence for relationships between the gene function and the physical and biological environments. One good example of such a non-functional gene is the gulonolactone oxidase (GLO) gene in primates, whose diet contains sufficient amounts of vitamin C. Given this improved diet, functional loss of the gene is less costly or even more beneficial than biosynthesis of vitamin C from *γ*-gulonolactone (Linster & Van Schaftingen 2007).

Genes often die, but whether or not such dead genes or pseudogenes can be fixed in a population in the context of the selectively relevant environment is a completely different matter. Conversely, it is possible to understand the biological implications of functional loss of genes in relation to palaeo-environments under which the pseudogenes arose and were evolutionarily accepted.

Examining the human and chimpanzee genomes *in silico*, Wang *et al*. (2006), Hahn & Lee (2006) and Hahn *et al*. (2007), the International Human Genome Sequencing Consortium (IHGSC 2001) and others (e.g. Torrents *et al*. 2003; Go & Niimura 2008) collectively enumerated more than 120 ‘HSPs’. Of these, 14 pseudogenes are polymorphic and the remaining ones have supposedly been fixed in the human population (table 3 and electronic supplementary material, table S2). However, since only the human and chimpanzee genomes were examined in most *in silico* studies, HSPs simply imply that they are disrupted by mutations in the human, but not in the chimpanzee. It is possible that some of these pseudogenes are also non-functional in other primates. In addition, these HSPs may include processed pseudogenes, truly functional genes that are misclassified as pseudogenes or pseudogenes without functional orthologues in non-human primates. We exclude all of these as HSP candidates.

**Table 3.** Examination of human specific pseudogenes (HSPs). (Criteria: a, the presence of closely related paralogues with sequence divergences of less than 10%; b, the presence of pseudogenes in non-human Catarrhini; c, processed pseudogenes; d, misclassified as pseudogenes; e, these pseudogenes are actually absent in the genome of either humans or non-human Catarrhini.)

		criteria^2^					
fixed candidates	no.^1^	a	b	c	d	e	no. of HSPs
T-cell receptor genes	4	0	0	0	0	0	4
olfactory receptor genes	53	10	5	0	1	12	25
taste receptor genes	2	1	1	0	0	0	0
other genes	48	9	18	5	6	7	9^3^
subtotal	107	20	24	5	7	19	38
polymorphic candidates	14	4	1	2	0	1	8
total	121	24	25	7	7	21	46

^1^The number of HSP candidates thus far identified.

^2^The five criteria (a to e) for the exclusion as HSPs are not mutually exclusive and there are six genes that are excluded by two different criteria.

^3^The nine HSPs are CMAH (Hayakawa *et al*. 2006), GLRA4 (IHGSC 2001), MBL1 (Wang *et al*. 2006), MYH16 (Stedman *et al*. 2004), ZNF850 (Wang *et al*. 2006), S100A15 (Hahn *et al*. 2007), SIGLEC13 (Angata *et al*. 2004), TDH (Edgar 2002), and KRT41 (Winter *et al*. 2001). See electronic supplementary material, table S2 for detail.

Perhaps more importantly, many HSPs identified thus far belong to multi-gene families. If there exist any closely related copies (paralogues) of a given pseudogene in the human genome, the functional loss of a copy is likely to be selectively neutral and to have nothing to do with the environment. To exclude this case too, we set an operational cut-off value of nucleotide substitutions *k*_c_ between a candidate pseudogene and a functional paralogue. Namely, wherever there exists a closely related functional paralogue with *k*_c_ ≤ 0.1 in the human genome, we exclude such a *trivial* pseudogene from the HSPs considered in our study.

The application of the above criteria to 107 fixed pseudogenes has left only 25 olfactory receptor (OR) pseudogenes and 13 other pseudogenes (table 3). The latter group of pseudogenes comprises four T cell receptors (TCR), CMAH, GLRA4, MBL1, MHY16, SIGLEC-13, TDH, KRT41 and two other less characterized genes. An immediate consequence is that the number of fixed HSPs is much smaller than previously claimed. This substantial reduction results, in part, from the inaccurate/incomplete genome database in non-human primates or the presence of closely related duplicated genes in the human genome or both and, in part, from the absence of orthologues in non-human primate genomes.

From the observation that the total 38 pseudogenes have been fixed in the human population, the overall fixation rate is 5–6 per genome per millon years or 2.2 × 10^−10^ per locus per year if the human genome contains 25 000 loci. We note, however, that the fixation rate differs considerably from one gene family to another. Large multi-gene families such as OR and TCR appear to have evolved with high rates. On the other hand, even apparently unique genes such as CMAH and TDH have also lost their functions. We tried to date the functional loss of seven unique genes to the exclusion of ZNF850P with a highly repetitive motif as well as SIGLEC13 that is completely deleted from the human genome (Angata *et al*. 2004). Of particular interest are the functional losses of GLRA4 and TDH, which occurred in this order, since both are involved in glycine metabolism or glycine transmittance and glycine acts as a neuro-transmitter in the mammalian central nervous system.

Because the number of authentic HSPs is discouragingly small, the interspecies differences between humans and chimpanzees cannot be entirely attributed to the functional loss of genes. In this respect, we have compared gene expression profiles in the skin of humans and chimpanzees and found that there are about 180 gene loci at each of which the human skin expresses greater than 100 times more transcripts than the chimpanzee skin or vice versa (data not shown). Although our experiment with microarray analyses are not exhaustive for other tissues and organs, we are inclined to agree with the supposition of Zuckerkandl & Pauling (1965), who proposed, ‘many phenotypic differences may be the result of changes in the patterns of timing and rate of activity of structural genes rather than of changes in functional properties of the polypeptides as a result of changes in amino-acid sequence.’ Functional loss of genes is certainly one extreme case of regulatory changes, but some other changes at the expression level appear to have played more important roles in human evolution.

## 5. Perspectives

When we initiated our studies reviewed in this article, only a limited number of pertinent DNA sequences were available. This situation has changed dramatically during the last two decades, followed by various innovations in theoretical and computational methods. Furthermore, genome-wide comparisons in large samples within and among species will soon offer new insights into significant evolutionary problems. One hundred and fifty years ago, Darwin (1859) eloquently concluded in *The Origin*:

Thus, from the war of nature, from famine and death, the most exalted object which we are capable of conceiving, namely, the production of the higher animals directly follows. There is grandeur in this view of life, with its several powers, having been originally breathed by the Creator into a few forms or into one; and that, whilst this planet has gone cycling on according to the fixed gravity, from so simple a beginning endless forms most beautiful and most wonderful have been and are being evolved. (Darwin 1859, p. 459)

To us, this ending is echoed in Brenner's (1991) remark that ‘because we have no direct access to the processes of evolution and can only study its contemporary products and relics of the past, it is here that the creative imagination plays an important role in the scientific endeavour.’ However, at the deepest level of the contemporary products, we have abundant informational *relics* at hand that surely would substantiate Darwin's thesis.
